# Implementation of Robotic Assistance in Pancreatic Surgery: Experiences from the First 101 Consecutive Cases

**DOI:** 10.3390/jcm10020229

**Published:** 2021-01-11

**Authors:** Lea Timmermann, Matthias Biebl, Moritz Schmelzle, Marcus Bahra, Thomas Malinka, Johann Pratschke

**Affiliations:** Department of Surgery, Charité–Universitätsmedizin Berlin, Corporate Member of Freie Universität Berlin, Humboldt-Universität zu Berlin, and Berlin Institute of Health, Augustenburger Platz 1, 10551 Berlin, Germany; lea.timmermann@charite.de (L.T.); matthias.biebl@charite.de (M.B.); moritz.schmelzle@charite.de (M.S.); marcus.bahra@charite.de (M.B.); johann.pratschke@charite.de (J.P.)

**Keywords:** robotic assisted surgery, pancreatic surgery, pancreaticoduodenectomy

## Abstract

Robotic assisted minimally invasive surgery has been implemented to overcome typical limitations of conventional laparoscopy such as lack of angulation, especially during creation of biliary and pancreatic anastomoses. With this retrospective analysis, we provide our experience with the first 101 consecutive robotic pancreatic resection performed at our center. Distal pancreatectomies (RDP, N = 44), total pancreatectomies (RTP, N = 3) and pancreaticoduodenectomies (RPD, N = 54) were included. Malignancy was found in 45.5% (RDP), 66.7% (RTP) and 61% (RPD). Procedure times decreased from the first to the second half of the cohort for RDP (218 min vs. 128 min, *p* = 0.02) and RPD (378 min vs. 271 min, *p* < 0.001). Overall complication rate was 63%, 33% and 66% for RPD, RPT and RDP, respectively. Reintervention and reoperation rates were 41% and 17% (RPD), 33% and 0% (RTP) and 50% and 11.4% (RPD), respectively. The thirty-day mortality rate was 5.6% for RPD and nil for RTP and RDP. Overall complication rate remained stable throughout the study period. In this series, implementation of robotic pancreas surgery was safe and feasible. Final evaluation of the anastomoses through the median retrieval incision compensated for the lack of haptic feedback during reconstruction and allowed for secure minimally invasive resection and reconstruction.

## 1. Introduction

Laparoscopic-assisted pancreatic surgery was initially only used for diagnostic purposes or palliative interventions like bypass procedures. In 1994, the first laparoscopic partial pancreaticoduodenectomy (LPD) was described [1]. In the following, several centres approached LPD. However, the results remained controversial. Some studies showed LPD to be either less safe or without display of the expected advantages like a shortened in-hospital stay or reduced blood loss compared to open surgery [2,3,4]. Other studies, on the other hand, confirmed comparable rates of perioperative morbidity and mortality [5] a reduction of length of stay [6,7], or even an advantage in oncologic outcome [8]. Overall, the general limitations of laparoscopy in the setting of complex reconstructions prevail, and therefore, robotic assistance arouses attention in pancreatic surgery after its implementation. It allows a three-dimensional, magnifiable view, up to seven degrees-of-freedom [9] and automatically reduces tremor transmission [10]. Additionally, the learning curve is shorter compared to laparoscopically assisted procedures [11,12,13,14].

Nevertheless, the main burdens of pancreatic surgery, including postoperative pancreatic fistula (POPF), postoperative pancreatic haemorrhage (PPH) and insufficiency of the implemented pancreatico-enteric or biliary anastomosis all apply to laparoscopic and robotic-assisted pancreatic surgery alike. As an important role is ascribed to the variable parenchymal texture of the gland, establishment and evaluation of the pancreatico-enteric anastomosis require a meticulous haptic examination for accomplishing adequate tactile feedback. Therefore, in addition to a high-level of laparoscopic skills, extensive experience in open pancreatic surgery seems indispensable for a successful implementation of a minimally invasive pancreatic surgery program [15]. The main aims of laparoscopic as well as robotic-assisted surgery, in general, are reduction of in-hospital stays and enhanced recovery while providing comparable or reduced complication rates. However, the value of a treatment option is not only measured by complication rates but also by its oncologic outcome, including relevant parameters such as lymph node harvest or R0-resection rates.

With this analysis, we describe the implementation process of robotic-assisted pancreatic surgery in our centre and provide the data of our first 101 consecutive cases undergoing robotic-assisted pancreatic resections.

## 2. Materials and Methods

### 2.1. Data Collection and Exclusion Criteria

Data of patients who underwent either distal pancreatectomy (DP), total pancreatectomy (TP) or partial pancreaticoduodenectomy in a robotic-assisted procedure in the time between October 2017 and December 2019 were included in this analysis. All data were collected within the CARE-Study (surgical assistance by robotic support; originally Chirurgische Assistenz durch Robotereinsatz, ethical approval code E/A4/084/17; (DRKS00017229)). Procedures with conversion laparotomy before completion of resection were excluded from further analysis. Early conversion laparotomy was necessary in four patients due to tumour extent exceeding a safe minimally invasive approach, due to a bleeding complication in one patient and due to pneumoperitoneum related ventilation issues in two patients.

### 2.2. Perioperative Course

Patients were admitted to our surgical ward at least one day before surgery. The concept of enhanced recovery after surgery (ERAS) had been applied within the study period. Preoperative assessment included computed tomography or magnetic resonance imaging with contrast agents as well as if indicated, chest imaging and endosonography. Physical examination, basic laboratory testing, blood cell count and measuring of CEA and CA-19.9 alongside individual anesthesiological evaluation completed the preoperative assessment. Every case of suspected or confirmed malignancy was discussed in our specialised tumour board before and after surgery. All patients after PD and TP were admitted to the intensive care unit for at least one day. Patients undergoing DP were either admitted to intensive care or directly to the surgical ward depending on comorbidities as well as the surgical and anesthesiological course. Drainages were removed, if on POD3 lipase levels within the drainages were lower than three times the serum levels. Following PD with the implementation of a PG, a nasogastric tube remained at least until postoperative day five and was removed after a contrast swallow study confirmed regular gastric emptying. The ISGPS classifications for POPF, PPH and DGE, were applied [16,17,18]. Complications were classified according to the Clavien/Dindo classification [19].

### 2.3. Implementation Process and Procedures

**Figure 1:**
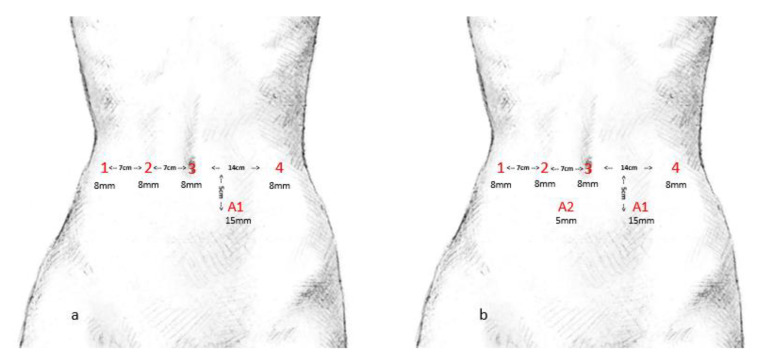
Port placement. (**a**) shows port placement for robotic assisted distal pancreatectomy. (**b**) shows port placement for robotic assisted pancreaticoduodenectomy and total pancreatectomy.

The same team of two surgeons performed all procedures with the DaVinci^®^ Xi surgical system (Intuitive Surgical Inc., Sunnyvale, CA, USA). Both were experienced in complex laparoscopic and open pancreatic surgery. Previous training consisted of computer-based lessons and intensive hands-on workshops. Figure 1 indicates the port placement, which is (a) suitable for RDP and (b) RPD and RTP.

Exclusion criteria for robotic assistance included (I) contraindication for creation of pneumoperitoneum (such as severe chronic obstructive lung diseases) and (II) multiple previous abdominal surgeries. Suspected extensive vessel involvements requiring additional resection (e.g., portal vein replacement) led to exclusion of the case and the patients underwent open surgery instead. In cases of underlying malignant disease or precancerous lesions, a standard lymphadenectomy was performed. Dissection of the pancreas was either done by electrocautery or a stapling device. In cases of underlying malignant disease or precancerous lesions located in the body or tail of the pancreas, a splenectomy and standard lymphadenectomy was performed. Patients with benign lesions received a spleen-preserving distal pancreatectomy with preservation of the splenic vessels. Stapler closure of the pancreas remnant was performed using linear staplers with a 60-mm black cartridge (EndoGIA™, Medtronic, Minneapolis, MN, USA) reinforced by a bioabsorbable mesh (SEAMGUARD^®^, W.L. Gore, Flagstaff, AZ, USA).

Early in the implementation phase, reconstruction following robotic-assisted PD (RPD) was carried out through the retrieval incision in the midline of the upper abdomen. Subsequently, we developed techniques for robotic-assisted hepaticojejunostomy and pancreatogastrostomy (PG). However, the retrieval incision remained essential for haptic reevaluation and, if indicated, correction of all anastomoses. In our centre, reconstruction following PD is in most cases carried out through a PG. During the implementation of a PG, suitable also for minimally invasive procedures, we developed a dorsal incision only PG for OPD that was subsequently also used for RPD. Every patient received at least one intra-abdominal drain (Degania Silicone Europe GmbH, Regensburg, Germany) to measure postoperative lipase levels and drain output in the postoperative course.

### 2.4. Statistical Analysis

Descriptive statistics and *t*-tests were used, and data were processed using SPSS version 25.0 (IBM, Armonk, NY, USA). *p* < 0.05 was considered statistically significant.

## 3. Results

This retrospective analysis included 101 consecutive patients, 44 of whom underwent DP, three underwent TP and 54 underwent PD. During the implementation process and this study period, 178 OPDs and no LPDs, 40 ODPs and six LDPs and 43 OTPs and no LTPs have been performed in our centre. Patient demographics are presented in Table 1.

Mean procedure time of the first 22 DPs was 217.9 min (minimum 142; maximum 353) with a standard deviation of 60 min. Mean procedure time of the second 22 DPs was 127.8 min (minimum 62; maximum 203) with a standard deviation of 34.6 min (*p* = 0.02).

**Figure 2:**
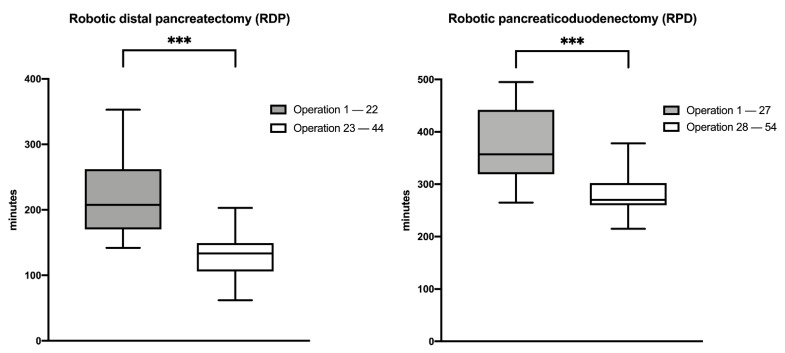
Development of procedure time. Decrease of procedure time comparing the initial and later series for RDP and RPD. *** *P* ≤ 0.001.

Mean procedure time of the first 27 PDs was 378.3 min (minimum 284; maximum 535) with a standard deviation of 72 min. Mean procedure time of the second 27 PDs was 276.1 min (minimum 215; maximum 378) with a standard deviation of 36.2 min (*p* < 0.001). Development of the procedure time for RDP and RPD are shown in Figure 2.

The overall complication rate for RPD was 63%, 48% were classified as major complications (Clavien–Dindo ≥ 3a). Following RTP, the overall complication rate was 33.3% (all classified as ≥ 3a) and following RDP 65.9% (56.8% ≥ 3a). Nine patients underwent re-operation following RPD (16.7%), three of them underwent completion pancreatectomy (one due to necrotizing pancreatitis of the pancreatic remnant, two due to bleeding complications), two of them underwent revision of implemented PG, one of them underwent revision of hepaticojejunostomy (HJ), one of them underwent revision due to trocar hernia and two patients underwent revision due to wound dehiscence or infection. Three patients following RDP underwent re-operation, two due to wound dehiscence and one due to colon perforation. Intraabdominal abscesses and persistent or recurrent fistula were treated with percutaneous or transgastric drainages. PG insufficiency was treated with re-operation in one case and transgastric drainage in the other cases. In cases of HJ-insufficiency either ERCP with stenting or PTCD was performed. All perioperative parameters are shown in Table 2.

In six of the first seven cases following RPD, reconstruction was carried out through the retrieval incision including PG, hepaticojejunostomy and gastroenterostomy. In the following, revision of the PG through the retrieval incision appeared to be necessary in four cases due to exceptionally soft gland texture after haptic re-evaluation. PG-insufficiency appeared in 6 patients, two of them following open reconstruction. Insufficiency of hepaticojejunostomy occurred in two cases, one of them following open reconstruction. There was no significant difference for perioperative complications between early and later cases, whereas the amount of full robotic procedures increased in the latest series. Conversion laparotomy was necessary due to bleeding from the splenic artery in one case of RDP. In contrast, conversion in the later phase of RPD was required in two cases, one due to bleeding complication and one due to technical issues. Table 3 indicates postoperative histopathology for all specimen. None of the patients undergoing RPD or RTP underwent neoadjuvant treatment and four patients underwent neoadjuvant chemotherapy prior to RDP.

## 4. Discussion

The learning curve for robotic assistance in pancreatic surgery is described to be quick and steep compared to laparoscopy. In our cohort, we achieved a significant decrease in OR time over the course of this series for both DP and PD. However, the learning process consisted not only of increased time savings but also of adoption of advantages as well as reaction to disadvantages of robotic assistance.

One of the main disadvantages of laparoscopic as well as robotic-assisted pancreatic surgery is the loss of direct haptic feedback, which is essential to examine the gland texture, the tumour extent and also the implemented anastomoses. For safety reasons, we therefore initially performed the restoration via PG, hepaticojejunostomy and gastrojejunostomy through the retrieval incision. After implementing a technique for robotic-assisted hepaticojejunostomy and PG, the retrieval incision remained essential for haptic re-evaluation of all anastomoses. In our opinion, this increases safety and circumvents the remaining uncertainty coming along with the missing haptic feedback in robotic-assisted procedures. This may serve as an explanation of the comparability of complication rates in early and later patients from our cohort for RPD while the amount of full robotic procedures increased in the latest phase of implementation. Other authors, however, describe significantly decreased overall complication rates and significant complication rates after the first series of 15 and 30 cases, respectively [20], which equals a substantial amount of complications encountered in the early adoption phase. Compared to our experiences in open pancreatic surgery, mortality is increased in RPD in this initial series (2.9% vs. 5.3% 30-day mortality). Further data are necessary to verify these findings in a larger cohort.

In cases of underlying malignancy, the tumour extent may limit the applicability of robotic-assisted procedures as well. In our cohort, early conversion laparotomy had become necessary due to tumour extent. Such borderline resectable cases, in our opinion, also require haptic evaluation to examine resectability in the first place thoroughly. Nevertheless, vascular resections during RPD have been described to be feasible after enclosed learning curve for RPD without additional vascular resection [21,22].

Despite all technical improvements during the last decades, the pancreatico-enteric anastomosis can still be referred to as the Achilles’ heel of current PD [23]. The superiority of neither reconstruction via pancreaticojejunostomy (PJ) nor PG in terms of complication rates and especially POPF incidence and severity for RPD is certain as, just like in open surgery, some studies advocate PG [24], while others prefer a PJ. PG-restoration via ventral gastrotomy, however, did not appear to be a technically feasible option for full robotic restoration. We, therefore, developed a dorsal-incision-only PG suitable for OPD and RPD alike.

Complication rates for robotic-assisted procedures in pancreatic surgery are comparable to open surgery [25]. Especially the main threats of modern pancreatic surgery, POPF, PPH and insufficiencies of pancreatico-enteric anastomoses seem equivalent [26,27]. In our cohort, we found a 30-day-mortality of 5.6% for RPD, and none of the patients following robotic-assisted DP died in the first 30 days after surgery. This has to be regarded with caution, as we aimed for careful patient selection, however, reflects in our opinion the cautious approach of our initial program, as these rates blend into other extensive experiences with open PD. We, therefore, found robotic assistance to be safe and feasible in our cohort. However, especially in the decision-making process of how far a minimally invasive approach can be pushed, we want to itinerate our impression of the surmount importance of extensive experience in both laparoscopy and open pancreatic surgery in order to safely embark on a robotic pancreas surgery program.

In our opinion, the structured step-by-step approach to the implementation of a robotic pancreas program with particular attention to a proper indication, port placement and reconstruction technique is essential. Considering the implementation of RPD in a two-step approach consisting of resection followed by reconstruction may be feasible.

Referring to the oncologic criteria, the amount of harvested lymph nodes is essential. Some studies suggest an increased number of harvested lymph nodes in minimally invasive procedures [28,29], whereas others did not find a difference between LPD, RPD and OPD [30]. The number of harvested lymph nodes in our cohort was comparable to other reports. Additionally, the rate of R0-resections is an important prognostic parameter, which is also said to increase with the use of robotic assistance [31]. As other authors already suggested, in our opinion, a structured training program, a sufficient volume and a close-knit quality assessment are essential for implementing a successful program for robotic-assisted pancreatic surgery [32].

A volume of at least 20 RPDs per year in a centre may maintain consistent training and complication rates [33]. However, despite insufficient training in a low-volume centre, cost-effectiveness decreases with low case numbers.

This descriptive analysis is limited to common biases, mainly due to its retrospective character and the deliberate patient selection in the investigated cohort. We are also well aware that, as long-term results following robotic-assisted pancreatic surgery are still missing, more studies are mandatory to evaluate robotic assistance as an individual prognostic parameter in the future.

## 5. Conclusions

Robotic assistance is a feasible and safe option in modern pancreatic surgery if attention is paid to the lack of haptic feedback in minimally invasive techniques. Additionally, with respect to complication rates, further studies are mandatory to evaluate its oncologic and long-term outcomes. For safety reasons, the indication should be made with appropriate caution, and conversion laparotomy should be used without reservation at any step of the procedure to prevent life-threatening complications.

## Figures and Tables

**Table 1:** Patient’s demographics.

Characteristics N (%)	Pancreaticoduodenectomy (N = 54)	Total Pancreatectomy (N = 3)	Distal Pancreatectomy (N = 44)
Sex N (%)			
**M**ale	32 (59.3)	1 (33.3)	22 (50)
**F**emale	22 (40.7)	2 (66.7)	22 (50)
Age (year)			
Minimum	27	41	22
Maximum	82	54	87
Mean	60.9	47.7	59.5
BMI (kg/m^2^)			
Minimum	19.7	23	18
Maximum	39.8	26.6	41.9
Mean	25.3	24.6	26.8
ASA-Score N (%)			
1	3 (5.9)	0	2 (4.8)
2	27 (52.9)	2 (66.7)	30 (71.4)
3	20 (39.2)	1 (33.3)	10 (23.8)
4	1 (2)	0	0 (0)
Malignancy N (%)	33 (61.1)	2 (66.7)	20 (45.5)

BMI: body mass index; ASA-Score: American Society of Anesthesiologists-Score.

**Table 2:** Perioperative parameters.

Characteristics N (%)	Pancreaticoduodenectomy (N = 54)	Total Pancreatectomy (N = 3)	Distal Pancreatectomy (N = 44)
Procedure Time (min)			
Minimum	215	258	62
Maximum	535	302	353
Mean	325.3	278	172.8
In-hospital stay (day)			
Minimum	3	10	5
Maximum	68	32	52
Median	15	11	11
ICU stay (day)			
Minimum	1	1	0
Maximum	55	6	12
Mean	6.6	2.6	1.9
Pancreatico-enteric anastomosis N (%)		None	None
Pancreaticojejunostomy	3 (5.6)		
Pancreatogastrostomy	51 (94.4)		
Overall complications N (%)	34 (63)	1 (33.3)	29 (65.9)
Clavien/ Dindo ≥3a N (%)	26 (48.1)	1 (33.3)	23 (52.3)
POPF N (%)			
Biochemical leakage	0 (0)	0 (0)	8 (18.1)
B	9 (16.7)	0 (0)	14 (31.8)
C	1 (1.9)	0 (0)	0 (0)
PPH N (%)			
A	3 (5.6)	0 (0)	2 (4.5)
B	4 (7.4)	0 (0)	3 (6.8)
C	4 (7.4)	0 (0)	0 (0)
SSI N (%)	5 (9.3)	0 (0)	1 (2.3)
DGE N (%)	10 (18.5)	0 (0)	1 (2.3)
Insufficiency pancreatico-enteric anastomosis N (%)	6 (11.1)	None	None
HJ-insufficiency N (%)	2 (3.7)	0 (0)	None
Pulmonary complications N (%)			
Pneumonia	5 (9.3)	0 (0)	3 (6.8)
Pulmonary embolism	0 (0)	0 (0)	2 (4.5)
Pleural effusion	4 (7.4)	0 (0)	3 (6.8)
Unplanned Re-Intubation	5 (9.3)	0 (0)	0 (0)
Intervention N (%)	22 (40.7)	1 (33.3)	22 (50)
Re-operation N (%)	9 (16.7)	0 (0)	5 (11.4)
30-day mortality N (%)	3 (5.6)	0 (0)	0 (0)

ICU: Intensive Care Unit; POPF: postoperative pancreatic fistula; PPH: postoperative pancreatic haemorrhage; SSI: surgical site infection; DGE: delayed gastric emptying; HJ: hepaticojejunostomy.

**Table 3:** Tumour histopathology.

Characteristics N (%)	Pancreaticoduodenectomy (N = 54)	Total Pancreatectomy (N = 3)	Distal Pancreatectomy (N = 44)
Histology N (%)			
PDAC	12 (22.2)	2 (66.7)	15 (34.1)
NET	1 (1.9)		4 (9.1)
Periampullary carcinoma	10 (18.5)		
Distal cholangiocarcinoma	7 (13.0)		
IPMN	10 (18.5)		8 (18.2)
Chronic pancreatitis	7 (13.0)		9 (20.5)
Other	8 (14.8)	1 (33.3)	8 (18.2)
T. N (%)			
T1	7 (23.3)	0 (0)	6 (31.6)
T2	13 (43.3)	1 (50)	10 (52.6)
T3	10 (33.3)	1 (50)	3 (15.8)
T4	0 (0)	0 (0)	0 (0)
N. N (%)			
N0	15 (48.4)	1 (50)	8 (47.1)
N1	5 (16.1)	0 (0)	9 (52.9)
N2	11 (35.5)	1 (50)	0 (0)
Lymph node harvest (N)			
Minimum	4	17	2
Maximum	28	29	44
Mean	16.5	23	12.8
V. N (%)			
V0	28 (93.3)	1 (50)	16 (84.2)
V1	2 (6.7)	1 (50)	3 (15.8)
L. N (%)			
L0	19 (63.3)	1 (50)	12 (63.2)
L1	11 (36.7)	1 (50)	7 (36.8)
G. N (%)			
G1	1 (3.3)	0 (0)	5 (29.4)
G2	19 (63.3)	1 (50)	4 (23.5)
G3	10 (33.3)	1 (50)	8 (47.1)
Pn N (%)			
Pn0	7 (24.1)	0 (0)	5 (33.3)
Pn1	22 (75.9)	1 (100)	10 (66.7)
R. N (%)			
R0	26 (83.9)	1 (50)	15 (75)
R1	5 (16.1)	1 (50)	5 (25)
Tumour diameter (mm)			
Minimum	8	40	1
Maximum	47	50	95
Mean	23.6	45	33.1

PDAC: pancreatic ductal adenocarcinoma; NET: neuroendocrine tumour; IPMN: intraductal papillary mucinous neoplasm; T.: local tumour state; N.: nodal state; V.: vessel invasion; L.: lymphatic vessel invasion; G.: Grading; Pn: perineural invasion; R.: resection state.

## Data Availability

The data presented in this study are available on reasonable request from the corresponding author.

## References

[1] Gagner M., Pomp A. (1994). Laparoscopic pylorus-preserving pancreatoduodenectomy. Surg. Endosc..

[2] van Hilst J., de Rooij T., Bosscha K., Brinkman D.J., van Dieren S., Dijkgraaf M.G., Gerhards M.F., de Hingh I.H., Karsten T.M., Lips D.J. (2019). Laparoscopic versus open pancreatoduodenectomy for pancreatic or periampullary tumours (LEOPARD-2): A multicentre, patient-blinded, randomised controlled phase 2/3 trial. Lancet Gastroenterol. Hepatol..

[3] Cuschieri A. (1996). Laparoscopic Pancreatic Resections. Semin. Laparosc. Surg..

[4] Dokmak S., Fteriche F.S., Aussilhou B., Bensafta Y., Levy P., Ruszniewski P., Belghiti J., Sauvanet A. (2015). Laparoscopic pancreaticoduodenectomy should not be routine for resection of periampullary tumors. J. Am. Coll. Surg..

[5] Nickel F., Haney C.M., Kowalewski K.F., Probst P., Limen E.F., Kalkum E., Diener M.K., Strobel O., Müller-Stich B.P., Hackert T. (2020). Laparoscopic Versus Open Pancreaticoduodenectomy: A Systematic Review and Meta-analysis of Randomized Controlled Trials. Ann. Surg..

[6] Palanivelu C., Senthilnathan P., Sabnis S.C., Babu N.S., Srivatsan Gurumurthy S., Anand Vijai N., Nalankilli V.P., Raj P.P., Parthasarathy R., Rajapandian S. (2017). Randomised clinical trial of laparoscopic versus open pancreatoduodenectomy for periampullary tumours. Br. J. Surg..

[7] Poves I., Burdio F., Morato O., Iglesias M., Radosevic A., Ilzarbe L., Visa L., Grande L. (2018). Comparison of Perioperative Outcomes Between Laparoscopic and Open Approach for Pancreatoduodenectomy: The PADULAP Randomized Controlled Trial. Ann. Surg..

[8] Peng L., Zhou Z., Cao Z., Wu W., Xiao W., Cao J. (2019). Long-Term Oncological Outcomes in Laparoscopic Versus Open Pancreaticoduodenectomy for Pancreatic Cancer: A Systematic Review and Meta-Analysis. J. Laparoendosc. Adv. Surg. Tech. A.

[9] Lefor A.K. (2019). Robotic and laparoscopic surgery of the pancreas: An historical review. BMC Biomed Eng..

[10] Leal Ghezzi T., Campos Corleta O. (2016). 30 Years of Robotic Surgery. World J. Surg..

[11] Zhang T., Zhao Z.M., Gao Y.X., Lau W.Y., Liu R. (2019). The learning curve for a surgeon in robot-assisted laparoscopic pancreaticoduodenectomy: A retrospective study in a high-volume pancreatic center. Surg. Endosc..

[12] Lu C., Jin W., Mou Y.P., Zhou J., Xu X., Xia T., Zhang R., Zhou Y., Yan J., Huang C. (2016). Analysis of learning curve for laparoscopic pancreaticoduodenectomy. J. Vis. Surg..

[13] Watkins A.A., Kent T.S., Gooding W.E., Boggi U., Chalikonda S., Kendrick M.L., Walsh R.M., Zeh H.J., Moser A.J. (2018). Multicenter outcomes of robotic reconstruction during the early learning curve for minimally-invasive pancreaticoduodenectomy. HPB.

[14] Napoli N., Kauffmann E.F., Perrone V.G., Miccoli M., Brozzetti S., Boggi U. (2015). The learning curve in robotic distal pancreatectomy. Updates Surg..

[15] Nota C.L., Zwart M.J., Fong Y., Hagendoorn J., Hogg M.E., Koerkamp B.G., Besselink M.G., Molenaar I.Q., The Dutch Pancreatic Cancer Group (2017). Developing a robotic pancreas program: The Dutch experience. J. Vis. Surg..

[16] Bassi C., Marchegiani G., Dervenis C., Sarr M., Abu Hilal M., Adham M., Allen P., Andersson R., Asbun H.J., Besselink M.G. (2017). The 2016 update of the International Study Group (ISGPS) definition and grading of postoperative pancreatic fistula: 11 Years After. Surgery.

[17] Wente M.N., Veit J.A., Bassi C., Dervenis C., Fingerhut A., Gouma D.J., Izbicki J.R., Neoptolemos J.P., Padbury R.T., Sarr M.G. (2007). Postpancreatectomy hemorrhage (PPH): An International Study Group of Pancreatic Surgery (ISGPS) definition. Surgery.

[18] Wente M.N., Bassi C., Dervenis C., Fingerhut A., Gouma D.J., Izbicki J.R., Neoptolemos J.P., Padbury R.T., Sarr M.G., Traverso L.W. (2007). Delayed gastric emptying (DGE) after pancreatic surgery: A suggested definition by the International Study Group of Pancreatic Surgery (ISGPS). Surgery.

[19] Dindo D., Demartines N., Clavien P.A. (2004). Classification of surgical complications: A new proposal with evaluation in a cohort of 6336 patients and results of a survey. Ann. Surg..

[20] Takahashi C., Shridhar R., Huston J., Meredith K. (2018). Outcomes associated with robotic approach to pancreatic resections. J. Gastrointest. Oncol..

[21] Beane J.D., Zenati M., Hamad A., Hogg M.E., Zeh H.J., Zureikat A.H. (2019). Robotic pancreatoduodenectomy with vascular resection: Outcomes and learning curve. Surgery.

[22] Shyr B.U., Chen S.C., Shyr Y.M., Wang S.E. (2020). Surgical, survival, and oncological outcomes after vascular resection in robotic and open pancreaticoduodenectomy. Surg. Endosc..

[23] Barreto S.G., Shukla P.J. (2017). Different types of pancreatico-enteric anastomosis. Transl. Gastroenterol. Hepatol..

[24] Giulianotti P.C., Gonzalez-Heredia R., Esposito S., Masrur M., Gangemi A., Bianco F.M. (2018). Trans-gastric pancreaticogastrostomy reconstruction after pylorus-preserving robotic Whipple: A proposal for a standardised technique. Surg. Endosc..

[25] Zureikat A.H., Moser A.J., Boone B.A., Bartlett D.L., Zenati M., Zeh H.J. (2013). 250 Robotic Pancreatic Resections Safety and Feasibility. Ann. Surg..

[26] McMillan M.T., Zureikat A.H., Hogg M.E., Kowalsky S.J., Zeh H.J., Sprys M.H., Vollmer C.M. (2017). A Propensity Score-Matched Analysis of Robotic vs Open Pancreatoduodenectomy on Incidence of Pancreatic Fistula. JAMA Surg..

[27] Magge D., Zenati M., Lutfi W., Hamad A., Zureikat A.H., Zeh H.J., Hogg M.E. (2018). Robotic pancreatoduodenectomy at an experienced institution is not associated with an increased risk of post-pancreatic hemorrhage. HPB.

[28] Wang S.E., Shyr B.U., Chen S.C., Shyr Y.M. (2018). Comparison between robotic and open pancreaticoduodenectomy with modified Blumgart pancreaticojejunostomy: A propensity score-matched study. Surgery.

[29] Marino M.V., Podda M., Gomez Ruiz M., Fernandez C.C., Guarrasi D., Gomez Fleitas M. (2020). Robotic-assisted versus open pancreaticoduodenectomy: The results of a case-matched comparison. J. Robot. Surg..

[30] Yan Q., Xu L.B., Ren Z.F., Liu C. (2020). Robotic versus open pancreaticoduodenectomy: A meta-analysis of short-term outcomes. Surg. Endosc..

[31] Peng L., Lin S., Li Y., Xiao W. (2017). Systematic review and meta-analysis of robotic versus open pancreaticoduodenectomy. Surg. Endosc..

[32] Moekotte A.L., Rawashdeh A., Asbun H.J., Coimbra F.J., Edil B.H., Jarufe N., Jeyarajah D.R., Kendrick M.L., Pessaux P., Zeh H.J. (2020). Safe implementation of minimally invasive pancreas resection: A systematic review. HPB.

[33] Jones L.R., Zwart M.J.W., Molenaar I.Q., Koerkamp B.G., Hogg M.E., Hilal M.A., Besselink M.G. (2020). Robotic Pancreatoduodenectomy: Patient Selection, Volume Criteria, and Training Programs. Scand. J. Surg..

